# Effects of Montmorency Tart Cherry and Blueberry Juice on Cardiometabolic and Other Health-Related Outcomes: A Three-Arm Placebo Randomized Controlled Trial

**DOI:** 10.3390/ijerph19095317

**Published:** 2022-04-27

**Authors:** Jonathan Sinclair, Lindsay Bottoms, Stephanie Dillon, Robert Allan, Gareth Shadwell, Bobbie Butters

**Affiliations:** 1 Research Centre for Applied Sport, Physical Activity and Performance, School of Sport & Health Sciences, Faculty of Allied Health and Wellbeing, University of Central Lancashire, Preston PR1 2HE, UK; sdillon@uclan.ac.uk (S.D.); rallan1@uclan.ac.uk (R.A.); gshadwell3@uclan.ac.uk (G.S.); bbutters2@uclan.ac.uk (B.B.); 2 Centre for Research in Psychology and Sport Sciences, School of Life and Medical Sciences, University of Hertfordshire, Hatfield AL10 9AB, UK; l.bottoms@herts.ac.uk

**Keywords:** tart cherry, blueberry, cardiovascular disease, blood pressure, metabolic health

## Abstract

The current study aimed to investigate the influence of tart cherry and blueberry juices on cardiometabolic and other health indices following a 20-day supplementation period. Forty-five adults were randomly assigned to receive tart cherry, blueberry, or a placebo, of which they drank 60 mL per day for 20 days. The primary outcome, which was systolic blood pressure, and secondary measures, including anthropometric, energy expenditure, substrate oxidation, hematological, diastolic blood pressure/resting heart rate, psychological wellbeing, and sleep efficacy, were measured before and after the intervention. There were no statistically significant differences (*p* > 0.05) for systolic blood pressure; however, total and LDL cholesterol were significantly improved with blueberry intake (pre: total cholesterol = 4.36 mmol/L and LDL cholesterol = 2.71 mmol/L; post: total cholesterol = 3.79 mmol/L and LDL cholesterol = 2.23 mmol/L) compared to placebo (pre: total cholesterol = 4.01 mmol/L and LDL cholesterol = 2.45 mmol/L; post: total cholesterol = 4.34 mmol/L and LDL cholesterol = 2.67 mmol/L). Furthermore, psychological wellbeing indices measured using the Beck Depression Inventory, State Trait Anxiety Inventory, and COOP WONCA improved statistically in the blueberry arm compared to placebo. Given the clear association between lipid concentrations and the risk of cardiovascular disease as well as the importance of psychological wellbeing to health-related quality of life, this investigation indicates that it could be an effective approach to assist in managing cardiometabolic disease.

## 1. Introduction

Cardiometabolic disease is now the principal cause of global mortality and healthcare expenditure [1]. Cardiometabolic syndrome itself is characterized by a range of symptoms, including hypertension, insulin resistance, atherogenic dyslipidemia, low high-density lipoproteins, high triglycerides, high adiposity, high body mass index, large waist-to-hip ratio, and poor glucose regulation [2,3]. Within the epidemiological literature, distinct pathophysiological markers of oxidative stress, nitric oxide, and inflammation have been cited as being the mechanistic indicators associated with the clinical presentation of cardiometabolic disease [4,5].

Pharmaceutical interventions represent the predominant treatment modalities for cardiometabolic conditions [6]. However, while these medications are efficacious in regards to the management of cardiometabolic disease, their cost-effectiveness remains ambiguous [7], and significant negative side-effects remain common [8]. Globally, it has been documented that 84% of adults over the age of 57 are prescribed at least one medication per day [9]. As such, it is clear that further natural and cost-effective remedies are required for cardiometabolic disease management and prevention [3].

Dietary practices are recognized as the most effective natural approach for the treatment of cardiometabolic disease such that most national/international medical organizations advocate improved nutrition for the prevention and management of this condition [10]. There is therefore a clear rationale for the implementation of dietary over pharmaceutical interventions, and indeed, Chiva-Blanch et al. [11] proposed that such approaches are likely to be more cost-effective and safer for the treatment and prevention of metabolic diseases. Diets rich in fruits and vegetables have been shown to attenuate the risk from cardiometabolic disease [12] although maintaining such approaches over a sustained duration has been shown to be difficult to accomplish [13]. Therefore, dietary supplements represent a potentially more appealing treatment and prevention modality.

Anthocyanins are abundant in dark-colored fruit and vegetable groups [14], and it is proposed that they may be able to confer significant improvements in cardiometabolic health [15]. Montmorency tart cherries, blueberries, strawberries, cranberries, and blackcurrants [16] in particular have been shown to possess high levels of anthocyanins [17] although the majority of peer-reviewed literature has focused on tart cherries. Supplementation of anthocyanin-rich tart cherries has been shown to improve oxidative stress [18,19] and inflammation [19,20,21], and blackcurrant supplementation was also shown to enhance fat-oxidation rates [22]. Improved fat oxidation during rest and physical activity is linked to long-term changes in body mass and composition allied with improvements in insulin sensitivity [23]. Therefore, an increased capacity to oxidize fat at rest and during moderate physical activity, initiated via anthocyanin-rich supplementation, may be advantageous for yielding improvements in body composition and insulin control, which is pertinent to cardiometabolic health. Importantly, the aforementioned anti-inflammatory, anti-oxidative, and substrate trafficking effects mediated through supplementation of anthocyanin-rich fruits appear to conveniently target the underlying chronic low-grade inflammation, pro-oxidant, and lipid-attenuating status that is central to cardiometabolic disease pathophysiology [24].

However, the findings from parallel randomized controlled trials exploring the effects of anthocyanin-rich fruit supplementation on cardiometabolic outcomes have yielded equivocal findings. Some studies exploring the effects of Montmorency tart cherry juice supplementation have shown no effect on cardiometabolic indices of blood pressure, triglycerides, insulin tolerance, or cholesterol [25,26,27,28], and some have revealed improvements in systolic blood pressure, total cholesterol, and low-density lipoprotein (LDL) cholesterol [4,6,29,30,31,32]. Studies exploring the efficacy of other anthocyanin-rich supplements present a similarly equivocal picture, with some demonstrating positive effects on cardiometabolic outcomes [33,34,35,36,37] and some showing no such effects [38,39,40,41].

### 1.1. Rationale

At the current time, there have yet to be any randomized intervention studies comparatively examining the efficacy of different anthocyanin-rich fruit supplements on cardiometabolic outcomes. With some food biochemical investigations showing that anthocyanin contents in dark fruits, such as blueberries, are as high or even greater than in tart cherries [17], further such investigations may be of both practical and clinical relevance.

### 1.2. Aim

The aim of the current study was to investigate the influence of 20 days of twice daily Montmorency tart cherry or blueberry juice supplementation on cardiometabolic and other health-related indices in healthy adults compared to placebo. The primary objective of this randomized trial is to examine the influence of the tart cherry and blueberry supplements on systolic blood pressure relative to placebo. Its secondary objectives are to assess if tart cherry juice and blueberry supplementation impacted other risk factors associated with and as a function of cardiometabolic disease.

### 1.3. Hypotheses

In relation to the primary outcome, it is expected that both Montmorency tart cherry and blueberry supplement groups will mediate significant reductions in systolic blood pressure compared to placebo, but no statistically significant differences will be observed between supplement groups. Furthermore, for the secondary outcomes, the Montmorency tart cherry and blueberry groups will produce improvements in cardiometabolic other health-related parameters compared to placebo, but there will be no statistically significant differences between the two supplement groups.

## 2. Materials and Methods

### 2.1. Study Design

**Figure 1:**
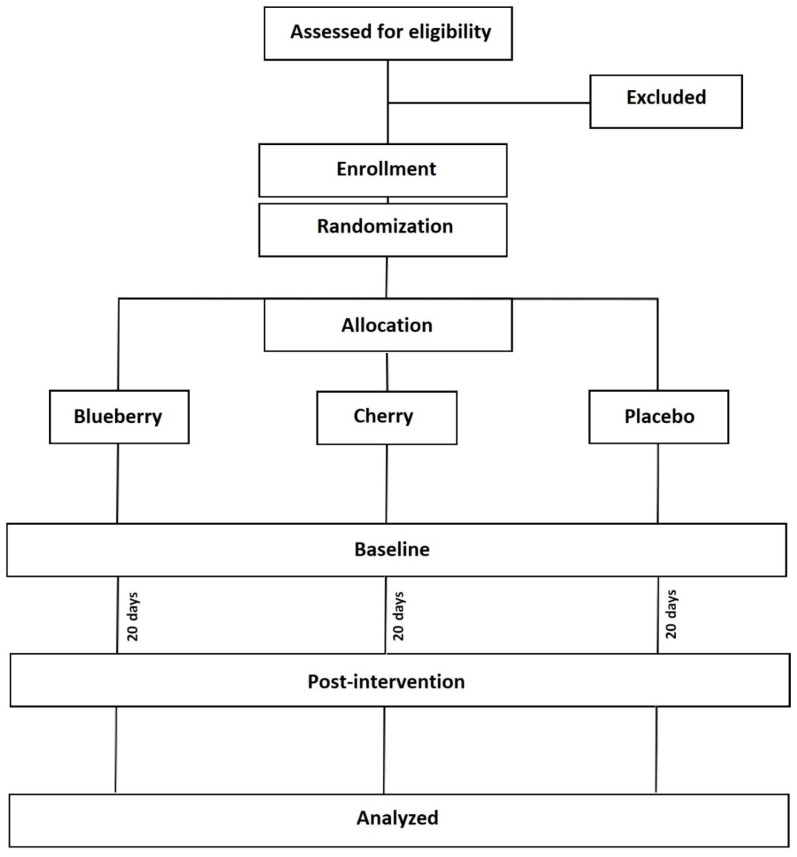
Consort diagram showing the study design.

This investigation represents a 20-day parallel, single-blind (blinded to participant), randomized placebo-controlled trial (Figure 1). The 20-day supplementation period was adopted in accordance with [28], and the protocol for this 3-arm randomized investigation has been previously published elsewhere [3] and was designed according to the updated guidelines for reporting parallel group randomized trials [42]. The study was registered prospectively (NCT04177238) and approved by an institutional ethical review board (HEALTH 0016).

After screening for eligibility and enrollment, participants were then randomized by a computer program (Random Allocation Software Version 1.0) to either (1) Montmorency tart cherry, (2) blueberry, or (3) placebo group. All experimental variables were assessed at (a) baseline (pre) and (b) after 20 days (post). In agreement with previous trials of cardiometabolic health, the primary outcome measure was the between-group difference in systolic blood pressure from baseline to post intervention [27]. Secondary outcome measures were between-group differences in anthropometric, energy expenditure and substrate oxidation (during rest and moderate intensity exercise), hematological, diastolic blood pressure/resting heart rate, psychological wellbeing, and sleep efficacy indices. All experimental testing took place in the morning in a ≥10 h fasted state, with participants having avoided strenuous exercise, alcohol, and nutritional supplements for 24 h and caffeine for 12 h prior to data collection [28].

### 2.2. Inclusion Criteria

- 18 years of age and above;
- Non-smoker;
- BMI  <  30;
- Able to give informed consent.

Exclusion criteria:

- Pregnancy;
- 65 years of age and above;
- Diabetes or any other metabolic/uncontrolled hypertensive conditions;
- Food allergies to cherries or blueberries;
- Habitual consumption of blueberries/cherries and/or blueberry/cherry products;
- Not regularly taking medication or antioxidant supplements.

### 2.3. Participants

Power calculations were performed for the primary outcome variable, i.e., the between-groups difference in systolic blood pressure. This showed that a total sample size of 45 was necessary to provide 80% power to detect a minimally important clinical difference (MCID) of 6 mmHg between groups [43], with a projected standard deviation of 5.5 mmHg in each group [44], accounting for a loss to follow up rate of 10%. Participants attended an eligibility, enrollment, and familiarization session prior to the commencement of formal data collection at the University of Central Lancashire. All participants provided informed consent in written form and completed a Par-Q screening form before taking part, in compliance with principles outlined in the declaration of Helsinki and the Oviedo Convention.

### 2.4. Dietary Intervention

After the conclusion of their baseline data collection session, participants were provided with either Montmorency tart cherry, blueberry, or placebo concentrate. Participants were required to consume 30 mL of supplementation diluted in 100 mL of water twice daily: once in the morning and again in the evening [27]. All supplementation was kept refrigerated throughout the 20 days. According to the manufacturer (ActiveEdge, Wintney, UK), a 30 mL dose of Montmorency tart cherry concentrate (Energy: 102 Kcal, carbohydrates: 25 g of which sugars: 18 g, protein: 1.10 g, and fiber: 2.6 g) is equivalent to approximately 320 mg of anthocyanins. Similarly, taking into account the manufacturers (ActiveEdge, Wintney, UK) guidelines, a 30 mL dose of blueberry concentrate (Energy: 103 Kcal, carbohydrates: 22 g of which sugars: 22 g, protein: 0.2 g, and fiber: 0.2 g) is equivalent to approximately 387 mg of anthocyanins.

Preparation of the placebo was undertaken in accordance with that outlined previously within the literature; this method of placebo preparation has been shown by previous intervention trials to provide an effective blinding strategy [45]. Placebo preparation involved mixing 100% un-flavored maltodextrin carbs (MyProtein, Cheshire, UK) into drinking water using a magnetic stirrer (Stuart Scientific, Staffordshire, UK) and stir bar (Fisher Scientific, Waltham, MA, USA). A total of 666 g of maltodextrin was added to water to create a liter of placebo concentrate, working out as 20 g of maltodextrin per 30 mL serving closely matching the Montmorency tart cherry or blueberry supplementation. Even amounts of red and black food coloring were added to match the color of the Montmorency tart cherry concentrate and even amounts of red, blue, and black coloring were utilized to match the color of the blueberry supplement. Either cherry or blueberry flavdrops (1 mL) (MyProtein, Cheshire, UK) were then added to match the required flavor. Irrespective of flavor, a 30 mL dose of placebo concentrate (100 Kcal, carbohydrates 25 g of which sugars: 0 g, protein: 0 g, and fiber 0 g) contained 0 mg of anthocyanins.

Throughout the study, the participants were encouraged to maintain their habitual diet and exercise routines and asked to refrain from consuming any multivitamin or antioxidant supplements [25]. For their post-intervention data collection session, all participants were asked to return any unused supplementation to determine the actual amount of supplement/placebo that was consumed (mL) and their % compliance. Furthermore, in order to explore the total quantity of supplementary ingested anthocyanins (mg), experimental average daily energy intake (Kcal/day) and supplementary average daily sugars (g/day) as well as the amount of supplementation that was consumed was multiplied by the anthocyanin, energy, and sugar contents established by the manufacturer. Finally, in order to examine blinding efficacy, each participant was asked whether they felt that they had been allocated to the supplement or placebo group at the conclusion of their post-intervention data collection session. In all three trial arms, loss to follow up was monitored as were any adverse events.

### 2.5. Data Collection

#### 2.5.1. Laboratory Visit Data

All measurements were undertaken at the University of Central Lancashire’s physiology laboratory (Preston, UK) and undertaken in an identical manner on two occasions, i.e., baseline and post intervention. The laboratories housed by the University of Central Lancashire are fully accredited by the British Association for Sport and Exercise Sciences, illustrating that they have undergone meticulous inspection and evidenced that all instrumentation is well maintained in terms of reliability, validity, and routine servicing; staff have the appropriate professional and vocational qualifications; and that the requisite operational procedures for health and safety are met.

#### 2.5.2. Anthropometric Measurements

Anthropometric measures of mass (kg) and stature (m) (without shoes) were used to calculate the body mass index (BMI) (kg/m^2^). Stature was measured using a stadiometer (Seca, Hamburg, Germany) and mass using weighing scales (Seca 875, Hamburg, Germany). In addition, body composition was examined using a phase-sensitive multifrequency bioelectrical impedance analysis device (Seca mBCA 515, Hamburg, Germany) [46], allowing percentage body fat (%) and fat mass (kg) to be quantified. Finally, waist circumference was measured at the midway point between the inferior margin of the last rib and the iliac crest and hip circumference around the pelvis at the point of maximum protrusion of the buttocks, without compressing the soft tissues [47], allowing the waist-to-hip ratio to be quantified.

#### 2.5.3. Energy Expenditure and Substrate Oxidation

Respiratory gases were collected using a gas analysis system (MetaLyser 3B system, Cortex Biophysic, Leipzig, Germany). The experimental laboratory was maintained using an air-conditioning system at a fixed ambient temperature of 20 °C. To quantify resting energy expenditure and substrate oxidation, participants laid supine for a period of 20 min, and data were extracted and averaged over the final 17 min [48]. Resting fat and carbohydrate oxidation rates (g/min) were quantified using the stoichiometric formulae outlined by Freyn, ref. [49] (Equations (1) and (2)), assuming negligible protein utilization. To quantify resting metabolic rate (RMR) (kcal/day) the formula of Weir [50] was adopted (Equation (3)).

Carbohydrate (g/min) = (4.55 × VCO_2_) − (3.21 × VO_2_) (1)

Fat (g/min) = (1.67 × VO_2_) − (1.67 × VCO_2_) (2)

RMR (kcal/day) = [(3.941 × VO_2_) + (1.1106 × VCO_2_)] × 1440 (3)

In addition, carbohydrate- and fat-oxidation rates (g/min) and energy expenditure per minute (kcal/min) were also examined during moderate intensity physical activity. Participants walked on a treadmill (hp Cosmos Pulsar, Nussdorf, Germany) at a velocity of 4.5 km/h for a period of 6 min. This walking velocity has reliably been shown to correspond to moderate exercise intensities [51]. Data were averaged over the last minute of the 6 min test. Fat- and carbohydrate-oxidation rates (g/min) as well as energy expenditure (kcal/min) during the exercise test were quantified using stoichiometric formulae outlined by Jeukendrup and Wallis [52], specifically developed for the exercise intensity examined in this study (Equations (4)–(6)).

Carbohydrate (g/min) = (4.21 × VCO_2_) − (2.962 × VO_2_) (4)

Fat (g/min) = (1.695 × VO_2_) − (1.701 × VCO_2_) (5)

Energy expenditure (kcal/min) = (0.550 × VCO_2_) − (4.471 × VO_2_) (6)

#### 2.5.4. Hematological Testing

Capillary blood samples were also collected via finger-prick using a disposable lancet after cleaning with a 70% ethanol wipe. Capillary triglyceride, total cholesterol, and glucose levels (mmol/L) were immediately obtained using three handheld analyzers (MulticareIn, Multicare Medical, Arezzo, Italy) and capillary hemoglobin levels (g/L) using a single handheld analyzer (HemoCue, Ängelholm, Sweden). From these outcomes, LDL cholesterol (mmol/L) was firstly quantified using the Anandaraja et al. [53] formula with total cholesterol and triglycerides as inputs. In addition, high-density lipoprotein (HDL) cholesterol (mmol/L) was also calculated by re-arranging the Chen et al. [54] equation to make HDL the product of the formulae. Both of these approaches have been shown to have excellent similarity to their associated lipoprotein values examined using immunoassay techniques r = 0.948 − 0.970 [55,56] The ratios between total and HDL cholesterol and between LDL and HDL cholesterol levels were also determined in accordance with Millán et al. [55].

#### 2.5.5. Blood Pressure and Resting Heart Rate

Blood pressure (mmHg) and resting heart rate (beats·min^−1^) measurements were undertaken in an up-right seated position at the end of the above-described resting energy expenditure test. Both peripheral measures of systolic and diastolic blood pressure and resting heart rate were be measured via a non-invasive, automated blood pressure monitor (OMRON M2, Kyoto, Japan), adhering to the recommendations specified by the European Society of Hypertension [56]. Three readings were undertaken, each separated by a period of 1 min [57], and the mean of the last 2 readings used for analysis.

#### 2.5.6. Questionnaires

Sleep quality is diminished in patients with cardiometabolic disease [58], and intake of dietary polyphenols [59] and supplementation of Montmorency tart cherry has been demonstrated to enhance sleep quality and symptoms of insomnolence [60,61]. Therefore, general sleep quality was examined using the Pittsburgh sleep quality index (PSQI) [62], daytime sleepiness using the Epworth Sleepiness Scale [63] and symptoms of insomnolence via the Insomnia Severity Index [64]. These questionnaires were utilized co-operatively to provide a collective representation of sleep efficacy.

Furthermore, psychological wellbeing is lower in those with cardiometabolic disease [65], and a high intake of dietary polyphenols has been shown to enhance indices of psychological wellbeing [66]. Therefore, general psychological wellbeing was examined using the COOP WONCA questionnaire [67], depressive symptoms using the Beck Depression Inventory [68], and state/trait anxiety with the State Trait Anxiety Inventory (STAI) [69]. Once again, these scales were utilized conjunctively to provide a collective depiction of psychological wellbeing.

### 2.6. Statistical Analysis

All continuous experimental variables are presented as mean and standard deviations (SD). Comparisons between participant characteristics and all experimental variables were undertaken at baseline, as were the % compliance levels, experimental anthocyanins, experimental energy intake, and experimental sugars (g/day) between the groups using linear mixed models, with group modeled as a fixed factor and random intercepts by participants. All analyses of the intervention-based data were performed using on an intention to treat basis. To determine the effects of the intervention on all of the outcome measures, differences between the three groups were examined using linear mixed models with group modeled as a fixed factor and random intercepts by participants adopted, adjusted for baseline values modeled as a continuous fixed covariate. For linear mixed models the mean difference (*b*), t-value and 95% confidence intervals of the difference are presented. Effects sizes for all statistically significant comparisons were quantified using partial eta squared (η_P_^2^). Blinding efficacy was examined using a one-way chi-square (*Χ*^2^) goodness of fit test. Finally, changes from baseline to 20 days in the experimental parameters were used to create binary variables, i.e., improve/did not improve for each participant. Pearson chi-square tests of independence were also used to undertake bivariate cross-tabulation comparisons between the three trial groups, specifically to test differences in the number of participants who exhibited improvements in the experimental outcomes, the number lost to follow-up, and the number of adverse outcomes in each group. Probability values for all chi-square analyses in this trial were calculated using Monte-Carlo simulation. All analyses were conducted using SPSS v27 (IBM Inc., SPSS, Chicago, IL, USA), and statistical significance for all analyses was accepted as the *p* ≤ 0.05 level. In the interests of conciseness and clarity, only experimental variables that presented with statistical significance as a function of the intervention are presented in the results section.

## 3. Results

### 3.1. Baseline Characteristics

All of the experimental measurements were contrasted at baseline for the participants who completed the trial, and no significant differences between groups were found (*p* = 0.06–0.98—Table 1).

### 3.2. Loss to Follow Up, Compliance, Ingested Anthocyanins, and Adverse Events

**Figure 2:**
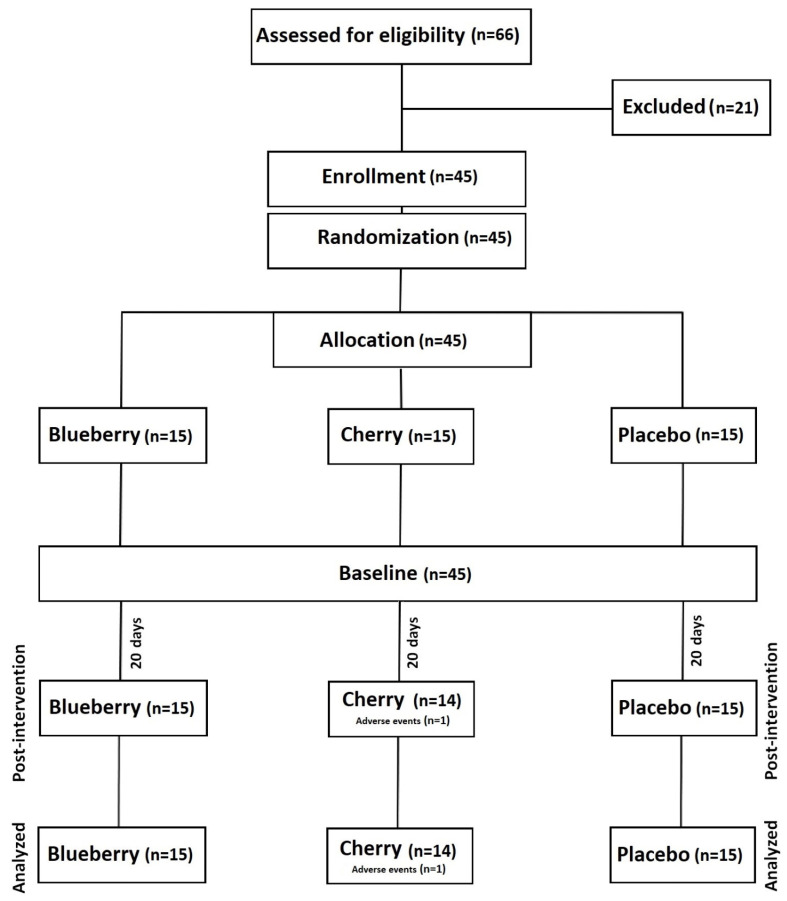
Consort diagram showing of participant flow throughout the study.

Total trial completion numbers in each group were cherry (*n* = 14), placebo (*n* = 15), and blueberry (*n* = 15), and number of adverse effects were cherry (*n* = 1), placebo (*n* = 0), and blueberry (*n* = 0). The chi-square tests were non-significant (*X*^2^ _(2)_ = 2.05, *p* = 0.36; *X*^2^ _(2)_ = 2.05, *p* = 0.36), indicating that there were no statistically significant differences between trial arms in either loss to follow up or adverse events (Figure 2).

There was no statistically significant difference in % compliance between the placebo and cherry (*b* = 1.47, (95% CI = −1.01–3.95), t = 1.21, *p* = 0.24), placebo and blueberry (*b* = 0.80, (95% CI = −1.70–3.30), t = 0.66, *p* = 0.52), or between cherry and blueberry groups (*b* = 0.67, (95% CI = −1.45–2.79), t = 0.64, *p* = 0.53). For supplementary anthocyanins, however, there were significant differences between the placebo and cherry (*b* = 6059.52, (95% CI = 5966.31–6152.73), t = 132.77, *p* < 0.001, η_P_^2^ = 0.98), placebo and blueberry (*b* = 14,759.66, (95% CI = 14,538.12–14,981.21), t = 136.06, *p* < 0.001, η_P_^2^ = 0.99), and between cherry and blueberry groups (*b* = 2640.62, (95% CI = 2351.08–2930.17), t = 18.63, *p* < 0.001, η_P_^2^ = 0.92). For supplementary daily sugars, there were significant differences between the placebo and cherry (*b* = 17.04, (95% CI = 16.78–17.30), t = 132.66, *p* < 0.001, η_P_^2^ = 0.98), placebo and blueberry (*b* = 41.95, (95% CI = 41.32–42.58), t = 136.57, *p* < 0.001, η_P_^2^ = 0.99), and between cherry and blueberry groups (*b* = 7.87, (95% CI = 7.05–8.69), t = 19.61, *p* < 0.001, η_P_^2^ = 0.93). Finally, for supplementary daily energy intake, there were significant differences between the placebo and cherry (*b* = 2.69, (95% CI = 0.27–5.12), t = 2.27, *p* = 0.031, η_P_^2^ = 0.26) and the placebo and blueberry (*b* = 8.65, (95% CI = 3.82– 13.49), t = 3.66, *p* = 0.001, η_P_^2^ = 0.31) but no differences between cherry and blueberry groups (*b* = 3.27, (95% CI = −0.92–7.45), t = 1.59, *p* = 0.121) (Table 2).

### 3.3. Blinding Efficacy

Of the 44 participants that completed the trial, 52% (*n* = 23) correctly identified their designated trial arm, and the chi-squared test was non-significant (*X*^2^ _(2)_ = 0.02, *p* = 0.89), indicating that an effective blinding strategy was adopted.

### 3.4. Anthropometric Measurements

No statistically significant differences (*p* > 0.05) in anthropometric parameters were found (Table 3).

### 3.5. Energy Expenditure and Substrate Oxidation

No statistically significant differences (*p* > 0.05) in energy expenditure and substrate oxidation parameters were found (Table 4).

### 3.6. Hematological Values

Adjusted for baseline, total cholesterol (*b* = 0.72, (95% CI = 0.19–1.24), t = 2.79, *p* = 0.009, η_P_^2^ = 0.21) and LDL cholesterol (*b* = 0.53, (95% CI = 0.09–0.97), t = 2.56, *p* = 0.020, η_P_^2^ = 0.17) were significantly reduced in the blueberry arm compared to placebo. Furthermore, adjusted for baseline glucose was significantly lower in the placebo (*b* = 0.61, (95% CI = 0.22–1.01), t = 3.18, *p* = 0.003, η_P_^2^ = 0.08) and cherry (*b* = 0.41, (95% CI = 0.10–0.72), t = 2.68, *p* = 0.012, η_P_^2^ = 0.11) arms compared to blueberry. Finally, adjusted for baseline, hemoglobin was significantly reduced in the placebo (*b* = 10.96, (95% CI = 1.61–20.32), t = 2.39, *p* = 0.023, η_P_^2^ = 0.16) arm compared to blueberry (Table 5).

For total cholesterol, the chi-square test was significant (*X*^2^ _(2)_ = 8.92, *p* = 0.012), and 80%, 86.7%, and 40% of participants exhibited improvements in the cherry, blueberry, and placebo groups, respectively. Similarly, for LDL cholesterol, the chi-square test was significant (*X*^2^ _(2)_ = 8.89, *p* = 0.011), and 60%, 86.7%, and 33.3% of participants exhibited improvements in the cherry, blueberry, and placebo groups, respectively. Finally, for triglycerides, the chi-square test was also significant (*X*^2^ _(2)_ = 6.01, *p* = 0.049), and 80%, 73.3%, and 40% of participants exhibited improvements in the cherry, blueberry, and placebo groups, respectively.

### 3.7. Blood Pressure and Resting Heart Rate

No statistical differences (*p* > 0.05) in blood pressure or resting heart rate were found (Table 6).

### 3.8. Questionnaires

Adjusted for baseline, Beck Depression Inventory (*b* = 1.90, (95% CI = 0.09–3.72), t = 2.14, *p* = 0.041, η_P_^2^ = 0.13), COOP WONCA (*b* = 0.31_,_ (95% CI = 0.06–0.56), t = 2.49, *p* = 0.019, η_P_^2^ = 0.17), state (*b* = 5.76, (95% CI = 1.04–10.49), t = 2.49, *p* = 0.018, η_P_^2^ = 0.17), and trait (*b* = 7.18, (95% CI = 1.05–13.32), t = 2.39, *p* = 0.023, η_P_^2^ = 0.16) anxiety scores were significantly reduced in the blueberry arm compared to placebo. Furthermore, adjusted for baseline, trait anxiety (*b* = 6.64, (95% CI = 0.40–12.89), t = 2.17, *p* = 0.038, η_P_^2^ = 0.15) scores were significantly reduced in the blueberry arm compared to cherry (Table 7).

## 4. Discussion

The current study aimed to investigate the influence of 20 days of twice daily Montmorency tart cherry or blueberry juice supplementation on cardiometabolic and other health-related indices in healthy adults compared to placebo. To date, this represents the first investigation to explore the effects of these supplementary interventions in a three-arm parallel placebo-controlled trial. The primary aim was to determine whether systolic blood pressure was improved as a function of these supplements, whereas the secondary aim(s) were to explore the effects of supplementation on other risk factors for cardiometabolic disease.

In relation to the primary outcome, the current investigation does not support our hypothesis in that there were significant reductions in systolic blood pressure in either the cherry of blueberry supplementation groups compared to placebo (Table 6). This result is in line with those of Lynn et al. [25], Desai et al. [28], and Kimble et al. [27], who showed that tart cherry supplementation had no effect on systolic blood pressure in healthy patients, while the current investigation also confirms a similar lack of efficacy for supplemental blueberry ingestion. It could therefore be speculated that in healthy individuals, arterial stiffness, which governs systolic blood pressure, is less responsive to short-term increases in anthocyanin intake via both tart cherry and blueberry supplementation. However, in healthy patients, Chai et al. [6] and Kent et al. [32] observed significantly lower systolic blood pressure in the first 3 h after ingestion, as did Desai et al. [29] and Keane et al. [4], in those with metabolic syndrome and early onset hypertension. Notably, Keane et al. [30] showed that plasma anthocyanin metabolites peak in the first two hours following ingestion with rapid clearance and return to basal within 4 h. This coincides with the aforementioned previously observed statistical reductions in postprandial blood pressure and suggests that the current and previous analyses adopting longitudinal rather than acute study designs may have missed the peak effects of tart cherry and blueberry supplementation. Importantly, as no statistically significant differences in blood pressure were observed, the current investigation lends further support to the concept that anthocyanin-rich supplementation mediates a transient rather than sustained attenuation of systolic blood pressure. Although physiologically important, the associated long-term clinical benefits of an acute reduction in blood pressure from both prophylactic and treatment standpoints has not yet been explored. Therefore, future analyses should seek to establish the enduring clinical efficacy of transient reductions in systolic blood pressure mediated through anthocyanin-rich supplementation.

Although no statistically significant differences in the primary outcome were evident, linear mixed-model and chi-square analyses support our hypothesis in that both total and LDL cholesterol were significantly improved in the blueberry arm compared to placebo (Table 5), and a larger number of participants experienced reductions in triglycerides in the cherry and blueberry groups. As no changes in HDL cholesterol were evident, it is clear that reductions in total cholesterol were mediated as a function of the corresponding attenuation in LDL values. Previous trials have shown that consuming tart cherry mediated statistical reductions in both total and LDL cholesterol in older patients [6] and those with metabolic syndrome [31] although this is the first investigation to show similar effects in healthy participants ingesting blueberry supplementation. It is proposed that the reductions in cholesterol mediated via the blueberry trial arm are a reflection of the statistically greater anthocyanin concentrations in this supplement (Table 2), lending support to the concept of a dose response to supplementary anthocyanins in cardiometabolic disease [70]. In relation to triglycerides, our observations concur with those in pathological patients [6], and the current investigation notably shows similar effects in healthy participants and efficacy also in those ingesting blueberry supplementation. Owing to the greater anthocyanin content in the blueberry supplement, it is proposed that the reductions in LDL cholesterol in this condition were mediated via the inhibition of plasma cholesteryl ester transfer protein (CETP). Several studies have indicated that CETP inhibition is a crucial mechanism for the attenuation of LDL cholesterol [71,72], and both human and animal analyses have shown that anthocyanins decrease plasma CETP activity [71,73]. Taking into account the long-standing and well-established association between lipid concentrations and the risk of cardiovascular disease [74], these observations may have considerable clinical relevance. While lipid-lowering pharmaceutics have been shown to exhibit a high level of efficacy, they are associated with significant side-effects [7], impose significant monetary restrictions on healthcare budgets, and contribute to the worldwide overreliance on prescription medications [8]. Therefore, the findings from the current trial lend support the concept that in particular blueberry supplementation may be important in the management of cardiometabolic disease.

Further regarding the improvements in total and LDL cholesterol shown in the blueberry trial arm, the current investigation also importantly showed that this supplemental condition was able to mediate statistical improvements in all indices of psychological wellbeing compared to placebo. This observation concurs with those of Khalid et al. [75], who showed improvements in mood state in both children and young adults ingesting a blueberry concentrate compared to placebo. Previous analyses have linked cardiometabolic disease to reduced levels of psychological wellbeing [65], so taking into account the aforementioned improvements noted in the blueberry trial arm, this observation makes intuitive sense. The mechanism responsible for the improvements in psychological wellbeing is not currently known and requires further consideration given the global incidence of depression and other psychological disorders [76]. There are several conceivable mechanisms that may explain our findings, including increased cerebral blood flow where cognitive and emotional control is located [77] and reduced monoamine oxidase activity causing increasing levels circulating monoamines, some of which are neurotransmitters associated with mood regulation [78]. Regardless, the observations from the current trial lend further support to the concept that blueberry supplementation may be important in the management of psychological disorders.

However, it is important to also acknowledge that despite the potentially exciting improvements in blood lipid and psychological wellbeing profiles in the blueberry trial arm, this supplement was associated with increased resting glucose values in relation to both the placebo and cherry trial arms (Table 5). It is apparent that this observation was caused by the increased sugar content (Table 3) in the blueberry supplement and the statistically greater daily sugar intakes in this arm compared to the others. This finding allied with the previously outlined reductions in blood lipids concurs with those of Chai et al. [6] with tart cherries. This observation is biologically interesting, as typically, increased blood glucose is met with corresponding increases in LDL cholesterol [79]. It is not within the scope of the biological measurements examined in this trial to accurately determine the mechanisms responsible for this finding. However, it can be speculated that the unique nature of the anthocyanin-rich blueberry supplementation may be responsible. Firstly, through the aforementioned CETP inhibition pathway as although potentially responsible for the attenuation of LDL cholesterol, animal models have shown strong correlations between CETP expression and bile acid signaling that may result in increased glucose disposal [80]. In addition, new information has shown that the influence of anthocyanins themselves on glucose and lipid metabolism in humans may be affected by their distinct chemical composition [81]. Blueberries are characterized by a greater number of hydroxyl groups [82] and belong to the malvidin variety of anthocyanins [83]. The malvidin group is associated with greater antioxidant capacity, which may explain their greater potential for improvements in dyslipidemia [84] although there is insufficient evidence concerning the effects of different anthocyanin compositions on blood glucose regulation. As such, it is important for further investigation to be conducted into the biological influence of anthocyanin chemical composition. Nonetheless, it is important to note that the mean fasting blood glucose values remained within normal ranges [85], and the long-term effects of elevated blood glucose levels remain unknown in healthy individuals. However, in patients with cardiometabolic conditions or diabetes mellitus characterized by poor glucose control, the findings from the current investigation do not currently support habitual utilization of this supplement despite the improvements in blood lipids. Therefore, it is important for future analyses to examine the longer-term effects of blueberry supplementation and to explore continuous blood glucose control as well as insulin and hemoglobin A1c indices in both healthy and pathological populations.

Overall, the current investigation exhibited a very effective level of blinding efficacy, a low number of adverse incidences, a high retention rate, very good compliance levels, as well as improvements in blood lipids and psychological wellbeing, predominantly in the blueberry trial arm. However, owing to the statistically greater mean daily sugar and associated kilocalorie intake (Table 3) in comparison to the cherry and placebo groups, in agreement with Kimble et al. [27], those seeking to utilize blueberry juice as a dietary supplement should utilize caution and seek to modify their daily dietary intake to account for the increase in daily Kcal. Furthermore, recent analyses have shown that some fruit phenolics constrain the formation of advanced glycation end products (AGE) [86] and thus mediate cellular and tissue impairment by damaging protein function and clearance [87]. Therefore, to better understand its potential biological effects, further exploration of the effects of blueberry juice should seek to examine the effects of this supplement on AGE formation. As with all research, this trial is not without limitations. Firstly, the experimental anthocyanin, energy, and sugar contents were reported according to the manufacturer’s guidelines, which, for anthocyanins in particular, have been shown to exhibit variability from sample to sample owing to differences in growing conditions [27]. While the current investigation observed positive effects of blueberry supplementation on cardiometabolic and psychological wellbeing indices, the mechanistic bases for these improvements was not elucidated. Therefore, future investigations should seek to explore and perhaps better utilize and exploit the mechanistic pathways of blueberry supplementation in order to improve health-related outcomes. Furthermore, as participants were randomized into their designated trial arms without consideration for their previous anthocyanin intake, a stratified random sampling approach should be adopted for future interventions exploring the effects of anthocyanin-rich fruits on cardiometabolic health indices. Finally, as many of the experimental variables are positively influenced by exercise, that physical activity was not monitored may serve as a limitation to this trial. Therefore, subsequent randomized interventions may seek to quantify physical activity throughout the intervention period via continuous actigraphy.

## 5. Conclusions

The current study aimed to investigate the influence of Montmorency tart cherry or blueberry juice supplementation on cardiometabolic, and other health-related indices compared to placebo. The current study did not support the primary hypothesis in that neither cherry or blueberry supplementation improved systolic blood pressure compared to placebo. However, the secondary hypothesis was supported in that 20 days of blueberry supplementation was able to mediate improvements in blood lipid concentrations and psychological wellbeing indices in relation to placebo. Given the clear and long-standing association between lipid concentrations and the risk of cardiovascular disease and the paramount importance of psychological wellbeing to health-related quality of life, the current investigation indicates that blueberry juice could represent a useful means to enhance cardiometabolic and psychological health. Future intervention trials and studies should consider exploring the longer-term effects of blueberry juice, the effects of increased supplemental sugar intake, as well as its efficacy in populations with cardiometabolic abnormalities at baseline.

## Figures and Tables

**Table 1:** Baseline characteristics of completed study participants.

	All		Placebo		Cherry		Blueberry	
	Mean	*SD*	Mean	*SD*	Mean	*SD*	Mean	*SD*
Age (years)	34.02	12.97	35.13	16.84	32.80	7.89	34.13	13.03
Mass (kg)	68.41	10.74	67.62	10.47	69.44	11.38	68.17	9.43
Stature (m)	1.68	0.09	1.67	0.10	1.69	0.10	1.68	0.08
BMI (kg/m^2^)	24.26	2.90	23.82	2.98	24.90	2.35	24.07	3.10
Sex (m/f)	24/20		8/7		7/7		9/6	

**Table 2:** Supplementary compliance and consumption throughout the intervention.

	Placebo		Cherry		Blueberry	
	Mean	*SD*	Mean	*SD*	Mean	*SD*
Amount consumed (mL)	1127	45	1136	34	1144	34
Compliance (%)	94	4	95	3	95	3
Experimental anthocyanins (mg)	0	8	12,119	366	14,760	435
Experimental energy intake (Kcal/day)	188	8	193	6	196	6
Experimental sugars (g/day)	0	0.	34	1	42	1

**Table 3:** Anthropometric measurements as a function of each trial arm.

	Placebo				Cherry				Blueberry			
	Pre		Post		Pre		Post		Pre		Post	
	Mean	*SD*	Mean	*SD*	Mean	*SD*	Mean	*SD*	Mean	*SD*	Mean	*SD*
Mass (kg)	67.62	10.47	69.27	12.55	69.44	11.38	69.62	9.94	68.17	9.43	66.46	7.87
Fat mass (kg)	15.55	5.07	16.61	6.07	17.97	6.18	17.85	5.36	17.02	5.18	15.83	4.11
BMI (kg/m^2^)	23.82	2.98	24.46	3.25	24.90	2.35	24.69	2.23	24.07	3.10	23.80	2.89
Body fat (%)	23.21	5.61	22.62	5.58	25.33	7.03	26.30	6.36	24.70	5.64	23.72	4.73
Waist circumference (m)	0.79	0.08	0.80	0.10	0.80	0.07	0.79	0.06	0.79	0.08	0.78	0.09
Waist:hip ratio	0.81	0.07	0.79	0.09	0.79	0.05	0.79	0.05	0.82	0.06	0.82	0.09

**Table 4:** Energy expenditure and substrate oxidation measurements as a function of each trial arm.

	Placebo				Cherry				Blueberry			
	Pre		Post		Pre		Post		Pre		Post	
	Mean	*SD*	Mean	*SD*	Mean	*SD*	Mean	*SD*	Mean	*SD*	Mean	*SD*
	Rest											
Carbohydrate oxidation (g/min)	0.23	0.06	0.29	0.11	0.23	0.05	0.24	0.05	0.21	0.06	0.21	0.08
Fat oxidation (g/min)	0.04	0.03	0.03	0.03	0.02	0.01	0.03	0.02	0.04	0.03	0.03	0.02
% Carbohydrate	69.24	16.23	70.26	17.82	71.01	8.67	69.66	12.63	68.65	18.15	68.55	21.53
% Fat	30.76	16.23	29.74	17.82	28.99	8.67	30.34	12.63	31.35	18.15	31.45	21.53
RMR (kcal/day)	1816.84	416.87	1797.67	580.28	1627.67	347.90	1683.43	306.60	1631.41	421.40	1638.71	388.42
	**Moderate intensity exercise**											
Carbohydrate oxidation (g/min)	0.40	0.20	0.52	0.26	0.69	0.26	0.68	0.24	0.45	0.26	0.45	0.25
Fat oxidation (g/min)	0.23	0.12	0.17	0.11	0.11	0.08	0.12	0.08	0.20	0.09	0.18	0.10
% Carbohydrate	75.18	22.27	76.73	23.01	72.00	20.10	71.59	19.23	73.12	25.46	72.23	26.71
% Fat	24.82	22.27	23.27	23.01	28.00	20.10	28.42	19.23	26.88	25.46	27.77	26.71
Energy expenditure (kcal/min)	3.72	0.88	3.71	0.84	3.91	0.78	3.87	0.66	3.71	0.87	3.49	0.75

**Table 5:** Hematological values as a function of each trial arm.

	Placebo				Cherry				Blueberry			
	Pre		Post		Pre		Post		Pre		Post	
	Mean	*SD*	Mean	*SD*	Mean	*SD*	Mean	*SD*	Mean	*SD*	Mean	*SD*
Cholesterol (mmol/L)	4.01	0.68	4.34	0.90	4.04	0.70	4.10	0.64	4.36	0.50	3.79 ^A^	0.58
LDL (mmol/L)	2.45	0.56	2.67	0.75	2.47	0.72	2.55	0.59	2.71	0.48	2.23 ^A^	0.45
HDL (mmol/L)	1.19	0.10	1.25	0.19	1.18	0.15	1.17	0.10	1.23	0.15	1.19	0.20
Total:HDL ratio	3.42	0.46	3.51	0.69	3.49	0.82	3.59	0.68	3.64	0.60	3.21	0.48
LDL:HDL ratio	2.11	0.40	2.17	0.62	2.14	0.75	2.23	0.64	2.29	0.55	1.91	0.45
Glucose (mmol/L)	4.71	0.80	4.36	0.64	4.60	0.60	4.54	0.42	4.55	0.56	4.93 ^A,B^	0.48
Triglycerides (mmol/L)	1.06	0.25	1.17	0.55	1.07	0.37	0.96	0.28	1.21	0.44	1.20	0.56
Hemoglobin (g/L)	141.40	18.77	136.05	12.58	140.07	11.30	142.45	13.17	145.37	12.38	146.93	13.18

Note: ^A^ = significant difference from baseline compared to placebo; ^B^ = significant difference from baseline compared to cherry.

**Table 6:** Blood pressure and resting heart rate measurements as a function of each trial arm.

	Placebo				Cherry				Blueberry			
	Pre		Post		Pre		Post		Pre		Post	
	Mean	*SD*	Mean	*SD*	Mean	*SD*	Mean	*SD*	Mean	*SD*	Mean	*SD*
Systolic blood pressure (mmHg)	123	20	120	16	118	9	119	9	120	12	122	14
Diastolic blood pressure (mmHg)	82	9	80	11	74	7	78	7	80	8	80	8
Resting heart rate (beats·min^−1^)	66	13	65	9	65	6	64	9	65	11	67	13

**Table 7:** Questionnaire measurements as a function of each trial arm.

	Placebo				Cherry				Blueberry			
	Pre		Post		Pre		Post		Pre		Post	
	Mean	*SD*	Mean	*SD*	Mean	*SD*	Mean	*SD*	Mean	*SD*	Mean	*SD*
Beck depression inventory	7.00	9.79	7.01	7.97	6.13	5.26	5.27	5.74	3.67	3.33	2.53 ^A^	2.92
COOP WONCA	1.95	0.58	2.06	0.45	1.92	0.55	1.93	0.54	1.88	0.36	1.71 ^A^	0.38
STAI state	32.60	10.76	35.87	11.10	33.47	7.68	32.73	8.39	30.67	9.55	28.87	6.83
STAI trait	40.20	10.66	40.73	11.43	36.87	8.72	39.20	8.67	39.33	9.98	33.07 ^A^	8.94
PSQI	4.47	2.20	4.40	2.06	5.67	2.23	5.27	1.87	5.40	3.07	5.07	3.61
Insomnia severity index	5.93	4.35	3.16	4.29	7.13	4.00	5.40	2.72	6.67	5.42	4.73	4.59
Epworth sleepiness scale	5.47	3.50	5.73	3.10	5.80	4.23	6.07	3.59	6.67	5.00	5.67	4.30

Note: ^A^ = significant difference from baseline compared to placebo.
